# Diagnostic value of lung ultrasound in interstitial lung disease associated with rheumatoid arthritis: Correlation with HRCT and differentiation of ILD subtypes

**DOI:** 10.1515/rir-2026-0012

**Published:** 2026-07-13

**Authors:** Asmaa Khalifa, Enas Khalifa, Tasneem Mohammed Bakheet, Mohamed Hamed, Mohamed Ramadan Izzaldin, Ahmed Elsaman

**Affiliations:** Department of Rheumatology and Rehabilitation, Sohag University Hospital, Sohag 82524, Egypt; Department of Chest diseases, Faculty of medicine, Sohag University Hospital, Sohag 82524, Egypt; Department of Public health and Community medicine, Sohag University Hospital, Sohag 82524, Egypt; Department of Radiology, Sohag University Hospital, Sohag 82524, Egypt; Department of Clinical Pathology, Al-azhar University Hospital, Assuit 71515, Egypt

**Keywords:** rheumatoid arthritis, interstitial lung disease, high resolution computed tomography, thoracic ultrasonography

## Abstract

**Objective:**

Rheumatoid arthritis (RA) is a systemic autoimmune disease in which interstitial lung disease (ILD) is a major extra-articular complication. Detection of RA-ILD is challenging because chest X-ray and pulmonary function tests have limited sensitivity, while high-resolution computed tomography (HRCT), although the reference standard, is constrained by cost and radiation exposure. Lung ultrasound (LUS) has therefore emerged as a practical bedside imaging tool. This study aimed to assess the diagnostic performance of LUS for RA-ILD using HRCT as the reference standard, examine its correlation with radiological and functional parameters, and evaluate its ability to distinguish ILD subtypes and severity stages.

**Methods:**

This prospective observational study included 86 RA patients with respiratory symptoms or auscultatory findings suggestive of ILD. All participants underwent clinical evaluation, CXR, PFTs, TUS, and HRCT. Sonographic parameters included B-line count, spacing, pleural line morphology, and lung sliding. HRCT findings were classified by ILD patterns, and the Warrick score was used to assess ILD severity. Receiver operating characteristic (ROC) analysis was performed to determine optimal B-line cutoffs.

**Results:**

Eighty-six RA patients were enrolled, with ILD confirmed by HRCT in 62 (72.1%). LUS demonstrated the highest diagnostic sensitivity (90.0%) among all modalities, outperforming CXR, PFTs, and auscultation. Diagnostic performance improved when stratified by HRCT pattern and disease severity, with higher AUCs for UIP (0.823), NSIP (0.816), and severe ILD (0.986). LUS B-line count showed a strong positive correlation with HRCT Warrick score (r = 0.933, p < 0.0001) and moderate negative correlations with forced vital capacity (FVC) and oxygen saturation. ROC analysis identified ≥ 10.5 B-lines as the optimal threshold for ILD detection (AUC = 0.766), achieving high sensitivity and moderate specificity.

**Conclusion:**

Lung ultrasound is an effective, accessible, and non-invasive screening modality for the detection of ILD in RA patients. Its integration into routine clinical practice can facilitate earlier diagnosis, assist in non-invasive differentiation of ILD subtypes, guide timely intervention, and improve patient outcomes. The identification of pattern- and severity-specific B-line cutoffs represents a novel contribution, extends existing evidence beyond a single universal threshold and highlights the added value of tailored ultrasound interpretation.

## Introduction

Rheumatoid arthritis is a progressive autoimmune disease that affects multiple systems in the body, with symptoms extending beyond the joints. Pulmonary complications are the most prevalent extra-articular manifestation, occurring in up to 40% of RA patients.^[1]^

Interstitial lung diseases (ILDs) include a group of conditions characterized by fibrosis and inflammation of the pulmonary interstitium. While ILD can occur as a secondary complication of RA, it can also result from medications used to manage RA. Numerous anti-inflammatory and biologic therapies have been linked to the development of ILD.^[2]^

Interstitial lung disease is a common and serious extra-articular manifestation of RA, playing a significant role in increasing both morbidity and mortality. It is the second most common cause of death in RA patients, surpassed only by cardiovascular complications.^[3]^

Despite its impact on patient prognosis, there is ongoing debate regarding the optimal timing, target population, and methods for screening this complication. ILD can emerge at any stage of RA progression. In over half of the cases, it develops after RA is diagnosed, usually within the first 5 to 10 years. Less commonly, ILD may appear at the onset of RA or even precede joint symptoms by months or years.^[4]^

Identification and evaluation of RA-ILD are critical to initiate treatment without delay, as patients may already have substantially reduced lung function by the time RA-ILD is diagnosed. High resolution computed tomography (HRCT) is regarded as the gold standard for detecting, diagnosing, and monitoring patients with ILD. Moreover, HRCT serves as the reference standard for assessing the diagnostic accuracy of other tools in RA-related ILD.^[4]^

When individuals with RA underwent screening for ILD HRCT, a notable proportion of subclinical disease was identified (ranging from 11.9% to 55.7%), highlighting that this complication is often underdiagnosed and this creates a rationale for a reproducible and radiation-free bedside tool for detection of potential ILD in RA.^[5]^

Thoracic ultrasound (TUS) is a bedside test that has shown promising results in RA-ILD and other ILD groups, including ILD related to systemic sclerosis (SSc-ILD), TUS can aid in detecting ILD by assessing B-lines, which serve as the ultrasound marker for pulmonary interstitial syndrome.^[6]^

Several studies have shown that TUS, due to its notable advantages such as cost-effectiveness, accessibility, and lack of radiation exposure, can serve as a valuable complementary tool in diagnosing (ILD). These benefits make it particularly useful when Chest X-ray (CXR) or HRCT is unavailable or not recommended, such as during pregnancy. While TUS has shown promise in detecting ILD in patients with rheumatoid arthritis (RA), its potential role in differentiating between ILD subtypes has not been fully established. Accordingly, the present study was designed to evaluate the diagnostic value of LUS in RA-associated ILD, assess its correlation with HRCT findings, and explore its capacity to distinguish between ILD subtypes.

## Materials and Methods

### Study Population

This observational prospective study was conducted at Sohag University Hospital and included 86 patients. The study protocol received approval from the Ethics Committee of the Faculty of Medicine, Sohag University, and informed consent was obtained from all participants.

Inclusion criteria consisted of adults (aged ≥ 18 years) diagnosed with RA based on the 2010 American College of Rheumatology/European League Against Rheumatism (ACR/EULAR) criteria,^[7]^ along with either: 1. Respiratory symptoms indicative of potential ILD; exertional dyspnea, chest discomfort (not explained by another condition), dry or productive cough with a duration longer than three months. 2. RA patients with dry “velcro-like” crackles on respiratory auscultation, even if asymptomatic.

Disease activity was assessed using the Disease Activity Score based on 28 joints with the erythrocyte sedimentation rate (DAS-28 ESR).^[8]^

### Exclusion Criteria

Patients with systemic autoimmune diseases other than RA, patients with cardiovascular diseases; or those with active infection or a history of COVID-19 infection; a history of or current cancer treated with chemotherapy, prior thoracic radiation therapy, or those with known ILD were not included in the study.

### Data Collection

All cases underwent comprehensive rheumatological and respiratory history taking and clinical examination at the rheumatology and pulmonology clinic, (b) CXR, (c) TUS, (d) Pulmonary function testing (PFT) and (e) High-resolution computed tomography which is used as the reference standard for interstitial lung diseases diagnosis,

### Thoracic Ultrasonography (TUS)

TUS was performed for all patients by a radiologist and a pulmonologist, both blinded to the patients’ clinical data, using a Mindray ultrasound system. Bio-Medical Electronics Co., Shenzhen, China. Both grayscale (B-mode) and time-motion (M-mode) techniques were employed to perform the assessments. Participants were examined while seated upright with their backs straight. The thorax was systematically scanned across the anterior, lateral, and posterior chest walls based on Volpicelli protocol.^[9]^ In each of the 12 zones, the transducer was placed vertically over an intercostal space, and the area was scanned to achieve the clearest view of the pleural line and detect any potential pathology.

The following parameters were evaluated: B-lines are described as long, distinct, laser-like, hyperechoic vertical artifacts that extend perpendicularly from the pleural line. They move in sync with respiration, originate at the pleural line, and extend to the full depth of the image without diminishing. B-lines are counted within a single lung intercostal space (LIS) between two ribs and are indicative of interlobular septal thickening.^[10]^

B-lines considered positive if three or more B-lines are present between two ribs in the longitudinal plane, a condition referred to as “alveolar-interstitial syndrome”. The distance between two adjacent B-lines near the pleural line is measured and recorded in millimeters. A measurement of B3 or B7 indicates that the distance between two B-lines is 3 mm or 7 mm, respectively.^[11]^

## Procedure for Counting B-lines

### Scanning Technique

Chest Zone Division According to Volpicelli Protocol: The thorax is divided into six regions per hemithorax (total of 12 zones):^[12]^

Anterior Zones

Upper: Between the clavicle and the second intercostal space. Lower: Between the second and fourth intercostal spaces.

Lateral Zones

Upper: Between the fourth and sixth intercostal spaces.

Lower: Below the sixth intercostal space.

Posterior Zones

Upper: Above the scapular spine.

Lower: Below the scapular spine.

*B-line Counting: (Total B Lines)*

The total B-line score for interstitial lung fibrosis is typically measured using a standardized scoring system based on the number and distribution of B-lines across different lung zones.^[13]^

1. Each scanned zone is scored based on the number of B-lines: 0 B-lines has score 0; 1–5 B-lines has score 1; 6–15 B-lines has score 2; > 15 B-lines or confluence has score 3.
2. The scores from all zones are summed to obtain the total B-line score.
3. The maximum possible score in 12-zone method → Max score= 36
4. Interpretation
  Mild ILF: Low total B-line score (*e.g*., < 10 – 15)
  Severe ILF: High Total B-line Score (> 20 – 25)

Lung sliding is characterized by the “to and fro” dynamic movement of the lung along the pleural line during respiration.^[10]^

Pleural line abnormalities encompass irregularities, thickening, fragmentation, and the presence of subpleural nodules. Pleural line irregularity manifests as a disruption of the smooth linear pattern and hyperechogenic pleural morphology. A pleural line is typically classified as thickened if it measures 2.4 mm or more, though some studies propose a threshold of 2.8 mm.^[14]^

An inter-observer reliability analysis for LUS:- inter-observer agreement was excellent for quantitative B-line counts (ICC = 0.91) and almost perfect for the presence or absence of B-lines per zone (*κ* = 0.97). Agreement for pleural-line abnormalities was moderate (κ ≈ 0.50).

Pulmonary function tests (PFT): PFT were conducted using a spirometer with computer processing (Jaeger Master Screen Diffusion, GmbH, Hochberg, Viasys Healthcare, Germany). A restrictive ventilatory defect was defined by spirometric criteria, where the FEV1/FVC ratio exceeded 70% of the predicted value, but FVC was less than 80% of the predicted value.^[15]^

High resolution computed tomography (HRCT): It was performed using a Toshiba Alexion 16-slice CT scanner. The predominant pattern observed on the HRCT scan was documented and classified as reticular, ground-glass, cystic, nodular, mosaic, honeycombing, or a combination of these patterns. An HRCT scan showing areas of ground-glass opacity, reticulation, or signs of established fibrosis (such as traction bronchiectasis, honeycombing, or noticeable volume loss) can serve as a positive reference standard for diagnosing, with specific disease patterns as: usual interstitial pneumonia (UIP), non-specific interstitial pneumonia (NSIP).^[16]^

The Warrick score is a semi-quantitative system used to assess the severity and extent Of ILD based on HRCT findings. It evaluates specific radiological features and assigns scores to quantify pulmonary involvement.

### Scoring Method

Severity score: This component assesses five HRCT features, each graded based on severity: Ground-glass opacities: Score 1; Irregular pleural margins: Score 2; Septal or sub-pleural lines: Score 3; Honeycombing: Score 4; Subpleural cysts: Score 5.

The severity score ranges from 0 to 15, with higher scores indicating more severe disease.

Extent Score: This evaluates the distribution of each feature across lung segments; The extent score also ranges from 0 to 15: 1 to 3 segments involved: Score 1; 4 to 9 segments involved: Score 2; More than 9 segments involved: Score 3.

The total Warrick score is the sum of the severity and extent scores, ranging from 0 to 30. Interpretation of Warrick Score: 0–7: Mild ILD; 8–15: Moderate ILD; > 15: Severe ILD.^[17]^

### Statistical Analysis

Data analysis was performed using SPSS version 25.0 (IBM Corp., Armonk, NY). Continuous variables were presented as mean ± standard deviation, while categorical variables were expressed as frequencies and percentages. The chi-square test & Fisher’s Exact Test were used to compare categorical variables, and independent *t*-tests & Mann-Whitney *U* test was performed for continuous data. Correlations between Warrick score and other diagnostic parameters were done. ROC was done to choose the diagnostic cutoff point of B lines. A *p*-value of < 0.05 was considered statistically significant. Sensitivity and specificity are key metrics for evaluating the diagnostic accuracy of a test. Sensitivity measures the TUS test’s ability to correctly identify RA-ILD in patients who truly have the disease (HRCT positive), while specificity reflects its ability to correctly exclude RA-ILD in patients without the disease (HRCT negative).

## Results

### Baseline Demographic and Clinical Characteristics of the Study Cohort

The study involved 86 patients, the mean age of the participants was (47 + 13) years, with 72 of them being female (83.7%). Regarding smoking status, 46.5% of patients were non-smokers, 14.0% were active smokers, and 39.5% reported passive smoking exposure. The mean body mass index (BMI) was 28.06 ± 4.87 kg/m^2^. The mean Disease Activity Score (DAS28) was 3.8 ± 0.8. A majority of the patients (65.1%) were classified as having moderate disease activity. Rheumatoid factor (RF) was positive in 88.4% of patients, while Anti-cyclic citrullinated peptide (anti-CCP) antibodies were positive in 41.9% of the patients (Table 1).

**Table 1:** Demographic and clinical features of the studied population, n = 86

Characteristics Gender	Male	Count 14	% 16.3
	Female	72	83.7
Age (yr)	Mean ± SD	47 ± 13	
BMI	Mean ± SD	28.06 ± 4.87	
Smoker	Non smoker	40	46.5
	Active	12	14.0
	Passive	34	39.5
DAS-28 ESR score	Mean ± SD	3.8 ± 0.8	
DAS-28 ESR	Remission	8	9.3
	Low	14	16.3
	Moderate	56	65.1
	Highly active	4	4.7
	Very highly active	4	4.7
RF	Negative	10	11.6
	Positive	76	88.4
Anti-CCP	Negative	50	58.1
	Positive	36	41.9
Respiratory symptoms	Asymptomatic	46	53.5
	Symptomatic	40	46.5
O2 saturation	Mean ± SD	92 ± 3	

BMI, body mass index; DAS-28 ESR, disease activity score based on 28 joints with the erythrocyte sedimentation rate; RF, rheumatoid factor; Anti-CCP, Anti-cyclic citrullinated peptide.

### Clinical and Demographic Characteristics of RA Patients With and Without ILD

Out of 86 RA patients assessed, ILD was detected in 62 patients (72.1%) by HRCT. Patients with ILD were significantly older than those without ILD (mean age 49 ± 13 *vs*. 42 ± 12 years, *P* = 0.03). DAS28 scores were higher among ILD patients compared to those without ILD, but this difference was not statistically significant (*P* = 0.175). However, when categorized, a higher proportion of ILD patients exhibited moderate to very high disease activity compared to non-ILD patients (65.1% *vs*. 18.6%), approaching statistical significance (*P* = 0.054). RF, anti-CCP, and methotrexate dose did not differ significantly between groups. Respiratory symptoms and lower oxygen saturation were significantly more common in the ILD group (Table 2).

**Table 2:** Association between patient characteristics and the presence of ILD by HRCT, n = 86

Characteristics No.		N0 ILD (*n* = 24)		ILD (*n* = 62)		*P* ^*^
		No.	%	No.	%	
Age (yr)	Mean ± SD	42 ± 12		49 ± 13		0.03
	Median (IQR)	42 (36:53)		50 (38:55)		
Gender	Male	4	4.7	10	11.6	0.90
	Female	20	23.3	52	60.5	
DAS-28 ESR	Remission	2	2.3	6	7.0	0.054
	Low	8	9.3	6	7.0	
	Moderate	14	16.3	42	48.8	
	Highly active	0	0.0	4	4.7	
	Very highly active	0	0.0	4	4.7	
DAS-28 ESR score	Mean ± SD	3.6 ± .7		3.9 ± 0.8		0.175
	Median (IQR)	3.7 (3.0 ± 4.3)		4 (3.5:4.3)		
Disease duration (yr)	Mean ± SD	6 ± 3		9 ± 7		0.053
	Median (IQR)	6 (4:9)		7 (4:13)		
Rheumatoid factor	Negative	4	4.7	6	7.0	0.40
	Positive	20	23.3	56	65.1	
Anti-CCP	Negative	14	16.3	36	41.9	0.90
	Positive	10	11.6	26	30.2	
MTX dose	Mean ± SD	17.7 ± 9.4		18.8 ± 8.7		0.30
(mg/week)	Median (IQR)	17.5 (10.9:25)		25 (12.5:25)		
Resp symptoms	Asymptomatic	22	25.6	24	27.9	<0.0007
	Symptomatic	2	2.3	38	44.2	
O2 saturation	Mean ± SD	96 ± 2		91 ± 2		<0.004
	Median (IQR)	97 (95: 98)		90 (90:92)		

Data presented as number and percentage from total. ^*^P values were calculated using Pearson Chi-square test, Fisher’s exact test, independent *t*-test, or Mann–Whitney *U* test, as appropriate. Highlighted values are significant. ILD, interstitial lung disease; HRCT, high-resolution computed tomography; DAS-28 ESR, disease activity score based on 28 joints with the erythrocyte sedimentation rate; RF, rheumatoid factor; Anti-CCP, Anti-cyclic citrullinated peptide.

### HRCT and Thoracic Ultrasound Findings in RA-ILD Patients

**Figure 1:**
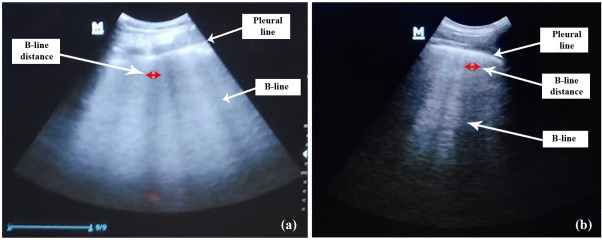
Lung ultrasound images demonstrating typical B-line patterns in RA-associated interstitial lung disease: (a) Multiple coalescent vertical hyperechoic artifacts (B-lines) with narrow inter–B-line spacing (B3), consistent with a NSIP pattern. (b) Multiple vertical hyperechoic artifacts (B-lines) with wider inter–B-line (B7), consistent with a UIP pattern. NSIP: nonspecific interstitial pneumonia; UIP: usual interstitial pneumonia.

HRCT predominantly demonstrated reticular opacities, followed by ground-glass opacities, while honeycombing was observed in 25% of cases. The mean Warrick score was 8.5 ± 5.3 (median 7, IQR 4–12), indicating mainly mild-to-moderate disease severity, with the UIP pattern more frequently identified than NSIP. On lung ultrasound, B-lines were detected in 80.6% of patients, with a mean total count of 18.9 ± 7.4. The B7 pattern was present in 58.1% and the B3 pattern in 41.9%. Pleural thickening was common (93.5%), while pleural irregularities (45.2%), subpleural lesions (19.4%), and diminished lung sliding (22.6%) were less frequent. B-line distance showed a strong positive correlation with the UIP pattern (*r* = 0.674, *P* < 0.001) and a negative correlation with the NSIP pattern (*r* = –0.674, *P* < 0.001), suggesting distinct sonographic profiles corresponding to HRCT patterns as shown in (Table 3) and (Figure 1).

**Table 3:** Combined HRCT and Thoracic Ultrasound (LUS) Findings in RA-ILD Patients (n = 62)

Finding	HRCT Results	LUS Results
ILD Patterns	UIP: 36 (58.1%)	B line distance correlation^*^
	NSIP: 26 (41.9%)	UIP: *r* = 0.674 (*P* < 0.0001)
		NSIP: *r* = -0.674 (*P* < 0.001)
Severity Scores	Warrick Score: 8.5 ± 5.3	B-line Count: 18.9 ± 7.4
	Mild: 30 (48.3%)	B-lines Present: 50 (80.6%)
	Moderate: 22 (35.5%)	
	Severe: 10 (16.2%)	
Parenchymal	Reticular: 28 (45.2%)	B-line Distance:
Features	GGO: 20 (32.3%)	B7: 36 (58.1%)
	Honeycombing: 16	B3: 26 (41.9%)
	(25.8%)	Subpleural Lesions: 12 (19.4%)
	Nodular: 14 (22.6%)	
	Cystic: 12 (19.4%)	
	Mosaic: 8 (12.9%)	
Pleural Features	Thickening: 16 (25.8%)	Irregular Pleural Line: 28 (45.2%) Pleural Thickening: 58 (93.5%) Diminished Sliding: 14 (22.6%)

* *r* = Pearson correlation coefficient. ILD, interstitial lung disease; HRCT, high-resolution computed tomography; UIP, usual interstitial pneumonia; NSIP, nonspecific interstitial pneumonia; GGO, ground-glass opacity.

### Diagnostic performance of lung ultrasound compared with other modalities and across ILD subgroups; UIP and NSIP

**Figure 2:**
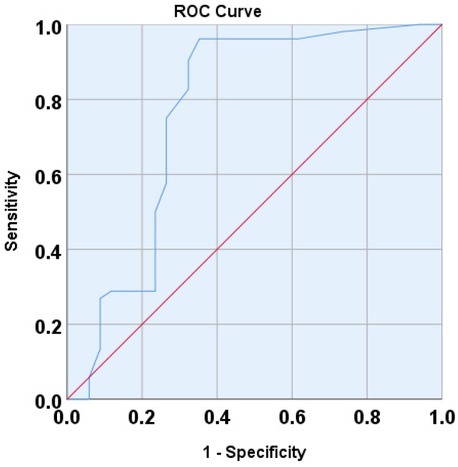
ROC curve for predilection of ILD with total B line. ROC: Forced vital capacity; ILD: interstitial lung disease.

Regarding diagnostic performance for ILD, LUS showed the highest sensitivity (90.0%) with a PPV of 84.3%, specificity of 61.5%, and NPV of 72.7%. CXR and PFT demonstrated lower sensitivity (32.3% and 25.8%, respectively), though with higher specificities (91.7% and 83.3%). Auscultatory crackles had poor sensitivity and specificity (35.5% and 38.3%, respectively), indicating limited diagnostic utility. Overall, LUS outperformed other diagnostic methods, supporting its utility as a screening tool for ILD in RA patients. ROC curve analysis showed that a total B-line cutoff of ≥10.5 achieved high sensitivity (90.4%) and moderate specificity (67.6%), with an overall diagnostic accuracy of 79% for identifying RA-ILD. The AUC was 0.766 (95% CI: 0.647–0.886; *P* < 0.0003), indicating moderate discriminative performance as shown in (Figure 2). Minor differences between sensitivity and specificity values reflect the use of ROC-derived optimal cutoffs versus binary diagnostic classification. Subgroup analyses revealed that the diagnostic performance of LUS varied according to HRCT pattern and disease severity. Pattern-specific evaluation showed improved discrimination for both UIP (AUC = 0.823) and NSIP (AUC = 0.816), with a lower optimal cutoff for UIP (≥ 10 B-lines) and a higher threshold for NSIP (≥ 15 B-lines). Notably, diagnostic accuracy increased progressively with disease severity, ranging from very good in mild ILD (AUC = 0.872) to outstanding in severe ILD (AUC = 0.986), with correspondingly higher optimal B-line cutoffs (≥ 11 for mild, ≥ 13 for moderate, and ≥ 24 for severe ILD). These findings indicate that while a universal cutoff is suitable for screening purposes, pattern- and severity-specific thresholds enhance the diagnostic precision of lung ultrasound and better reflect underlying disease burden as shown in (Table 4).

**Table 4:** Diagnostic performance of lung ultrasound compared with other modalities and across ILD subgroups

Analysis	Optimal Cutoff	AUC (95% CI)	Sensitivity	Specificity	PPV	NPV	Youden’s Index	*P*-value
Comparison with Other	Diagnostic Modalities	(*n* = 86)						
Thoracic Ultrasound	—	—	90.0%	61.5%	84.3%	72.7%	—	—
Chest X-ray	—	—	32.3%	91.7%	90.9%	34.3%	—	—
PFT (FVC)	—	—	25.8%	83.3%	80.0%	30.3%	—	—
Auscultatory Crackles	—	—	35.5%	38.3%	84.6%	33.3%	—	—
LUS Diagnostic Performance	by Subgroup							
Overall ILD detection	≥ 10.5 B-lines	0.766 (0.647–0.886)	90.4%	67.6%	—	—	0.580	<0.0003
UIP pattern	≥ 10 B-lines	0.823 (0.726–0.920)	84.0%	73.8%	—	—	0.578	<0.0001
NSIP pattern	≥ 15 B-lines	0.816 (0.718–0.914)	75.0%	79.0%	—	—	0.540	<0.0001
Mild ILD	≥ 11 B-lines	0.872 (0.780–0.964)	84.0%	81.1%	—	—	0.651	<0.0001
Moderate ILD	≥ 13 B-lines	0.928 (0.857–0.999)	94.1%	86.5%	—	—	0.806	<0.0001
Severe ILD	≥ 24 B-lines	0.986 (0.959–1.000)	100%	94.6%	—	—	0.946	<0.0001

PPV, positive predictive value; NPV, negative predictive value; PFT, pulmonary function test; ILD, interstitial lung disease; UIP, usual interstitial pneumonia; NSIP, nonspecific interstitial pneumonia.

### Correlation Between HRCT Severity (Warrick Score) and Clinical, Functional, and Sonographic Parameters

**Figure 3:**
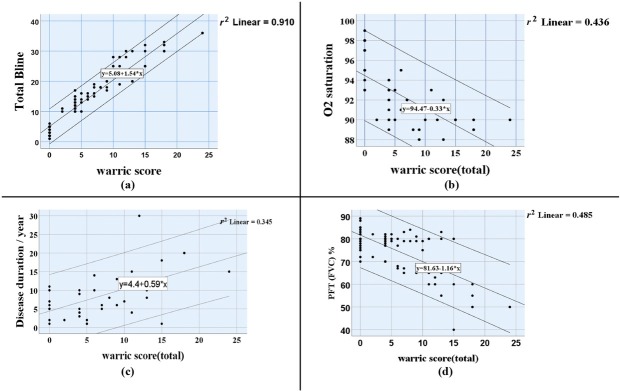
Correlations between Warrick score and disease characteristics in patients with RA-ILD. (a) Total B-line count, (b) Oxygen saturation, (c) Disease duration, and (d) Forced vital capacity (ROC, % predicted). Solid lines indicate linear regression, and shaded areas represent 95% confidence intervals. RA-ILD: rheumatoid arthritis–associated interstitial lung disease.

The Warrick score strongly correlated with B-line count (*r* = 0.933, *P* < 0.0001), and moderately with FVC (*r* = –0.685, *P* < 0.0001), oxygen saturation (*r* = –0.389, *P* = 0.002), and disease duration (*r* = 0.587, *P* < 0.0001), indicating that greater radiological severity was associated with reduced pulmonary function, lower oxygenation and longer disease duration (Table 5) (Figure 3).

**Table 5:** Correlation between Warrick score & B line numbers, FVC, Oxygen saturation and disease duration in ILD, n = 62

Variables	Warrick score	
	*r*	*P*
B line numbers	0.933	<0.0001
FVC	-0.685	<0.0001
Oxygen saturation	-0.389	0.002
Disease duration	0.587	<0.0001

FVC, forced vital capacity.

## Discussion

Although overall survival in rheumatoid arthritis has improved, patients with RAILD continue to experience significantly poorer outcomes than those without pulmonary involvement, highlighting the need for earlier detection using accessible imaging tools.^[18]^ Lung ultrasound has gained attention as an ILD screening tool in RA and systemic autoimmune diseases, detecting parenchymal alterations indirectly through characteristic artifacts rather than direct anatomical visualization.^[19]^

In this cohort, 60.5% of RA patients with ILD were women. This agrees with Santos-Moreno *et al*, who reported 78% female,^[19]^ but this contrasts with other studies, which have reported a male-to-female ratio of approximately 2:1.^[20]^ Regarding age, patients with RA-ILD had a mean age of 49 ± 13 years, which is lower than that reported by Kim *et al*. (65.8 ± 9.9 years) in a prospective RA-ILD cohort.^[21]^ This diference may reflect earlier recognition of ILD in our population. Nevertheless, the association between increasing age and RA-ILD underscores age as an important clinical marker for ILD risk in patients with rheumatoid arthritis.

In terms of disease activity, Sparks *et al*. reported an increased risk of RA-ILD in patients with elevated DAS28, supporting the association between systemic inflammation and pulmonary involvement.^[22]^ These findings align with the results of our study and suggest that controlling inflammation may influence RAILD. However, RoblesPérez *et al*. noted pulmonary progression despite improved joint symptoms,^[23]^ indicating factors beyond systemic inflammation contribute to lung involvement. Our findings showed no significant association between ILD and weekly methotrexate dose or RF/ antiCCP seropositivity, consistent with Abdelwahab *et al*. and RoblesPérez *et al*., who also reported no link between antiCCP levels or RA pharmacologic treatment and ILD development or progression.^[24]^

Early RA-ILD detection is challenging as initial symptoms (cough, fatigue, dyspnea) are nonspecific and may be masked by infection, medications, or joint-related immobility.^[16]^ A subset of patients remains asymptomatic, delaying diagnosis. In our study, 24 of 62 RA-ILD patients were asymptomatic, with dry inspiratory crackles incidentally detected during routine auscultation, prompting further evaluation.

Consistent with earlier findings, LUS demonstrated high sensitivity (90.4%) for RAILD at a cutof of ≥ 10.5 Blines (AUC 0.766).^[25]^ However, our optimal cutof is higher than the ≥ 5 Blines identified by Edith *et al*. (AUC 0.86).^[26]^ The variation in optimal B-line cutof values compared with previous studies likely reflects diferences in patient populations (*e.g*., higher fibrotic/UIP prevalence in our cohort), scanning protocols (including our comprehensive 14-zone protocol versus the 8-zone approach), and RAspecific factors such as pleural involvement and chestwall abnormalities which can generate incidental Blines.

The pattern-specific cutofs we identified (≥ 10 for UIP, ≥ 15 for NSIP) align with the histological underpinnings of these entities—UIP’s typically more focal, basal distribution versus NSIP’s difuse involvement requiring more extensive B-lines for detection. The exceptional performance in severe ILD (AUC 0.986, sensitivity 100%) corroborates findings by Wang *et al*. who noted near-perfect discrimination in advanced fibrosis stages.^[27]^ These collective observations suggest that pattern- and severity-specific cutoffs improved diagnostic performance, supporting the clinical use of tailored B-line thresholds rather than a single universal value.

In this study, LUS specificity was 61.5%, influenced by false positives. While some reports show lower specificity (< 40%)^[28]^ others align with our findings.^[11]^ These discrepancies may reflect population differences and the operator-dependent nature of LUS. In contrast, chest X-ray demonstrated low sensitivity for ILD despite high specificity versus HRCT, confirming its limited role as a standalone screening tool in early or subclinical RAILD.^[29]^

In our study, LUS demonstrated the highest sensitivity compared to PFT (25.8%), CXR (32.3%), and auscultation (sensitivity and specificity < 40%). Although CXR and PFT showed higher specificity (91.7% and 83.3%), their low sensitivity limited utility. These results align with VicenteRabaneda *et al*., where CXR and PFT also exhibited high specificity (98.1%, 98.3%) but markedly low sensitivity (2.5%, 8.7%), and auscultation showed lower accuracy (sensitivity 27.5%, specificity 77.3%) than LUS. This supports LUS as a sensitive, noninvasive tool for RAILD detection, especially where HRCT access is limited.^[11]^

In this study, LUS revealed extensive interstitial involvement, with Blines in 80.6% of patients higher than Vermant *et al*., where abnormalities were detected in about half. Our cohort showed more fibrotic features, such as pleural thickening and subpleural lesions. Pleural effusion was rare in both studies.^[30]^

Ancut *et al*. reported significant correlations between B-lines and HRCT fibrosis (*r* = 0.32) and FVC (*r* = –0.42),^[31]^ Ottaviani *et al*. found positive correlation with Warrick score (*r* = 0.836, *P* < 0.001) and negative with PFTs (*r* = −0.649, *P* < 0.001).^[32]^ Tüzü*n et al*. observed strong correlation with Warrick scores (*r* = 0.838).^[33]^ DiCarlo *et al*. and Gutierrez *et al*. similarly reported positive correlations with Warrick scores and negative with PFT parameters.^[34,35]^ Our results align with these findings, showing even stronger positive correlation with fibrosis severity (*r* = 0.933) and moderate negative correlation with FVC (*r* = –0.685), supporting LUS as a marker of interstitial involvement and functional impairment.

In our study, sonographic B-line distance showed strong positive correlations with the UIP pattern and negative correlations with the NSIP pattern on HRCT, indicating differing ultrasound profiles between subtypes. These findings align with Milena Adina *et al*., where a statistically significant difference was observed between UIP and NSIP groups (*P* < 0.001), suggesting LUS may aid in differentiating ILD subtypes in RA patients.^[36]^

This study has several limitations. The relatively small, single-center cohort may limit the generalizability of the findings. In addition, enrollment was restricted to RA patients with clinical suspicion of ILD, which may limit applicability to sub-clinical disease. Lung ultrasound is an operator-dependent technique; although standardized protocols were applied, variability cannot be completely excluded. Future multicenter studies with larger populations and double-blinded HRCT interpretation including inter-observer agreement analysis are warranted to further validate these results.

## Conclusion

This study demonstrates that lung ultrasound provides a reliable, non-invasive method for identifying RA-associated interstitial lung disease, demonstrating a strong correlation with HRCT findings. Beyond overall detection, our results indicate that lung ultrasound may also aid in differentiating ILD subtypes, as distinct B-line patterns in LUS, were associated with UIP and NSIP on HRCT. The identification of pattern- and severity-specific B-line cutoffs represents a novel contribution, extends existing evidence beyond a single universal threshold and highlights the added value of tailored ultrasound interpretation. These results support the role of LUS as a complementary imaging modality that can enhance risk stratification and clinical assessment in RA-ILD while potentially reducing reliance on repeated HRCT examinations and ultimately improve patient care by offering a bed-side, cost-effective, and radiation-free alternative for longitudinal assessment.
